# Meta-analysis of [^18^F]FDG-PET/CT in pulmonary sarcoidosis

**DOI:** 10.1007/s00330-024-10949-4

**Published:** 2024-07-23

**Authors:** Ryan Donnelly, Michael McDermott, Gerry McManus, Alessandro N. Franciosi, Michael P. Keane, Emmet E. McGrath, Cormac McCarthy, David J. Murphy

**Affiliations:** 1 St. Vincent’s University Hospital, Elm Park, Dublin, 4 Ireland; 2 University College Dublin, Belfield, Dublin, 4 Ireland

**Keywords:** Sarcoidosis, Pulmonary, 18F-FDG PET/CT, Prognosis, Diagnosis

## Abstract

**Background:**

18F-Fluorodeoxyglucose (FDG) PET/CT is emerging as a tool in the diagnosis and evaluation of pulmonary sarcoidosis, however, there is limited consensus regarding its diagnostic performance and prognostic value.

**Method:**

A meta-analysis was conducted with PubMed, Science Direct, MEDLINE, Scopus, and CENTRAL databases searched up to and including September 2023. 1355 studies were screened, with seventeen (*n* = 708 patients) suitable based on their assessment of the diagnostic performance or prognostic value of FDG-PET/CT. Study quality was assessed using the QUADAS-2 tool. Forest plots of pooled sensitivity and specificity were generated to assess diagnostic performance. Pooled changes in SUVmax were correlated with changes in pulmonary function tests (PFT).

**Results:**

FDG-PET/CT in diagnosing suspected pulmonary sarcoidosis (six studies, *n* = 400) had a pooled sensitivity of 0.971 (95%CI 0.909–1.000, *p* = < 0.001) and specificity of 0.873 (95%CI 0.845–0.920)(one study, *n* = 169). Eleven studies for prognostic analysis (*n* = 308) indicated a pooled reduction in pulmonary SUVmax of 4.538 (95%CI 5.653–3.453, *p* = < 0.001) post-treatment. PFTs displayed improvement post-treatment with a percentage increase in predicted forced vital capacity (FVC) and diffusion capacity of the lung for carbon monoxide (DLCO) of 7.346% (95%CI 2.257–12.436, *p* = 0.005) and 3.464% (95%CI -0.205–7.132, *p* = 0.064), respectively. Reduction in SUVmax correlated significantly with FVC (*r* = 0.644, *p* < 0.001) and DLCO (r = 0.582, *p* < 0.001) improvement.

**Conclusion:**

In cases of suspected pulmonary sarcoidosis, FDG-PET/CT demonstrated good diagnostic performance and correlated with functional health scores. FDG-PET/CT may help to guide immunosuppression in cases of complex sarcoidosis or where treatment rationalisation is needed.

**Clinical relevance statement:**

FDG-PET/CT has demonstrated a high diagnostic performance in the evaluation of suspected pulmonary sarcoidosis with radiologically assessed disease activity correlating strongly with clinically derived pulmonary function tests.

**Key Points:**

- *In diagnosing pulmonary sarcoidosis, FDG-PET/CT had a sensitivity and specificity of 0.971 and 0.873, respectively.*
- *Disease activity, as determined by SUVmax, reduced following treatment in all the included studies.*
- *Reduction in SUVmax correlated with an improvement in functional vital capacity, Diffusion Capacity of the Lungs for Carbon Monoxide, and subjective health scoring systems.*

## Introduction

Sarcoidosis is a multisystem inflammatory disease characterised by the growth of non-necrotising granulomas that can accumulate in virtually any organ in the body. The aetiology of the disease remains poorly understood, with a highly variable clinical course. It is estimated to occur in roughly 2 to 160 people per 100,000, with a varied geographic prevalence likely due to a combination of environmental factors and genetic susceptibility [1, 2]. Thoracic involvement affecting the mediastinal lymph nodes and lung parenchyma is the most common disease manifestation. Indeed, symptoms related to this are often the cause of the first presentation. Approximately 60 to 70% with pulmonary disease will see spontaneous resolution of symptoms before any intervention is implemented [3]. However, for patients where the disease persists, severity can range from asymptomatic nodal involvement to a progressive fibrotic process leading to eventual respiratory failure that is difficult to treat [4]. For this reason, the accurate and timely diagnosis of subclinical but active pulmonary sarcoidosis is important.

At present, the diagnosis of sarcoidosis is not standardized but is generally accepted based on three major criteria: (i) a clinically compatible picture with pathognomonic features, (ii) radiological evidence and (iii) a histological specimen demonstrating non-necrotising granulomatous disease [5]. The diverse spectrum of clinical presentations can make the diagnosis of sarcoidosis challenging for clinicians, particularly when disease activity can vary, and conventional markers are often inconclusive. CT is the standard diagnostic imaging test, with appearances ranging from an almost pathognomonic pattern of mediastinal and hilar nodal enlargement to more nonspecific nodal and pulmonary changes requiring further investigation [6]. Therefore, histological evidence is typically needed establish the final diagnosis.

Fluoro-2-deoxyglucose-18 (FDG) positron emission tomography (PET/CT) has emerged as a sensitive method of detecting sites of inflammation, sometimes before morphological changes are visible on anatomical imaging [7]. FDG PET/CT has an established role in cardiac sarcoidosis diagnosis and assessment of disease activity [8]. It is unclear at present, how FDG PET/CT correlates with pulmonary involvement, lung function and treatment response. To date, no meta-analysis or systematic literature review has been published on this topic. The aim of this meta-analysis of the current literature was to (i) assess the diagnostic performance of FDG-PET/CT for patients with suspected pulmonary sarcoidosis and (ii) determine the correlation between FDG-PET/CT and other clinically relevant biomarkers.

## Materials and Methods

### Data sources and literature search

PubMed, Science Direct, MEDLINE, Scopus, and CENTRAL databases were searched from inception until the end of September 2023 (R.D. and M.McD.). Studies evaluating the diagnostic performance and/or prognostic value of F-18 FDG PET for pulmonary sarcoidosis in association with clinical markers of lung function were the focus of our literary review. Studies meeting the criteria for pulmonary sarcoidosis were defined as those involving sarcoidosis impacting both the lung parenchyma and the mediastinal nodes. Search terms were kept purposely vague to capture as many studies as possible and avoid excluding patients with concomitant cardiac, pulmonary and/or systemic sarcoidosis involvement:

“Positron Emission Tomography Computed Tomography”[Mesh] OR “Positron-Emission Tomography”[Mesh]) AND “Fluorodeoxyglucose F18”[Mesh]) AND “Sarcoidosis”[Mesh].

### Study selection

Search results were entered into the systematic review software Covidence (Covidence systematic review software, Veritas Health Innovation, Melbourne, Australia). Duplicates were automatically removed by the Covidence software, and eligibility assessment was performed independently by two physicians (R.D. and M.McD.). Study titles were initially reviewed separately for inclusion, and any differences in physician decision were resolved by consensus discussion. Studies which did not present original data were excluded. In addition, small group case reports, review articles, books, opinion pieces and editorials were excluded. Literature was excluded if it was not published in English. Once initial title screening was performed, studies were retrieved for review and assessed further based on the details of their abstract and the eligibility criteria that we have outlined below.

### Diagnostic eligibility criteria and data extraction

All studies considered for inclusion in the diagnostic subgroup were required to assess histologically proven de-novo sarcoidosis patients prior to commencement of therapy. Patients were also accepted for inclusion if they were deemed to have sarcoidosis based on the American Thoracic Society/European Respiratory Society/World Association for Sarcoidosis and Other Granulomatosis Disorders (ATS/ERS/WASOG) criteria [5] (See Supplementary appendix). Publications which did not identify the presence of pulmonary sarcoidosis or used non-FDG PET tracers were excluded. Studies were examined and datapoints relating to the diagnostic accuracy of FDG-PET/CT including true positive/true negative/false positive/false negative or relevant sensitivity and specificity, were recorded, and tabulated.

### Prognostic study eligibility criteria and data extraction

All studies considered for inclusion in the prognostic subgroup were required to assess either histologically proven sarcoidosis or clinically diagnosed sarcoid according to the ATS/ERS/WASOG criteria [5]. Radiological assessment was performed via FDG-PET/CT, excluding non-FDG-PET/CT tracers. Studies were required to perform a comparative assessment of disease severity through either serial FDG-PET/CTs or reassessment of functional scores following treatment. Semi-quantitative measurements of metabolic activity (maximum standardized uptake value, SUVmax) in lung parenchyma and/or mediastinal stations were recorded. No exclusion was placed on the time since initial sarcoid diagnosis, or the type/duration of treatments received. Corresponding functional scores such as FVC and DLCO at the start and completion of treatment were recorded and correlated with initial disease severity as assessed radiologically.

### Quality and risk of bias assessment

Risk of bias assessment was carried out using the QUADAS-2 tool [9]. Two physicians performed this process independently and were blinded to each other’s evaluation. A structured list of ‘low concern’, ‘some concern’ and ‘high concern’ for risk of bias was generated in four main domains. Items included patient selection, index test used, reference standard, flow, and timing. When a full review was performed, any discrepancies in the risk of bias assessment were resolved via discussion. Results of this were then graphically displayed using the publicly available RobVis software [10].

### Statistical analysis

#### Diagnostic subgroup analysis

The diagnostic performance as declared in the 6 included studies was used to generate a pooled forest plot of sensitivity scores. A result for specificity was calculated from the 1 eligible study. Descriptive statistics including positive likelihood ratio (LR + ) and diagnostic odds ratio (DOR) were generated from these values. Statistical analysis was done using Excel (Microsoft Corp.) and SPSS 29.0.1.0 (IBM Corp.).

#### Prognostic subgroup analysis

SUVmax before and after treatment were recorded. A reduction in SUVmax was used as an indicator of disease response. All the recorded SUVmax scores were derived from published values in the mediastinum or lung parenchyma. The change in SUVmax (⍙SUVmax) following treatment in both the lung parenchyma and mediastinal stations was correlated with the change in functional vital capacity (⍙ FVC) and the diffusing capacity of the lung for carbon monoxide (⍙ DLCO). Pulmonary function scores were all reported as mean change in the percentage of predicted FVC or DLCO. Forest and funnel plots generated to assess the mean pooled effect size of SUVmax, FVC and DLCO. Radiological response to treatment, as assessed by SUVmax, was correlated with the change in pulmonary function using Pearson’s Coefficient (r). An r value of < 0.29 was scored as a weak correlation, 0.30–0.49 as a moderate correlation, and 0.50–1.00 as a strong correlation. In studies where discrete SUVmax and pulmonary function scores were provided for individual patients, a scatter plot relating discrete ⍙SUVmax to ⍙ FVC and ⍙ DLCO was created along with a line of best fit.

#### Quality-of-life analysis

Studies which provided quality-of-life (QOL) assessment scores were recorded along with mean standard deviation (SD). Short Form Health Survey Questionnaire (SF-36) mean scores were compared across studies and a mean change of > 10 was deemed significant. Statistical analysis was performed using Excel (Microsoft Corp.) and SPSS 29.0.1.0 (IBM Corp.).

## Results

### Literature search and selection of studies

**Fig. 1:**
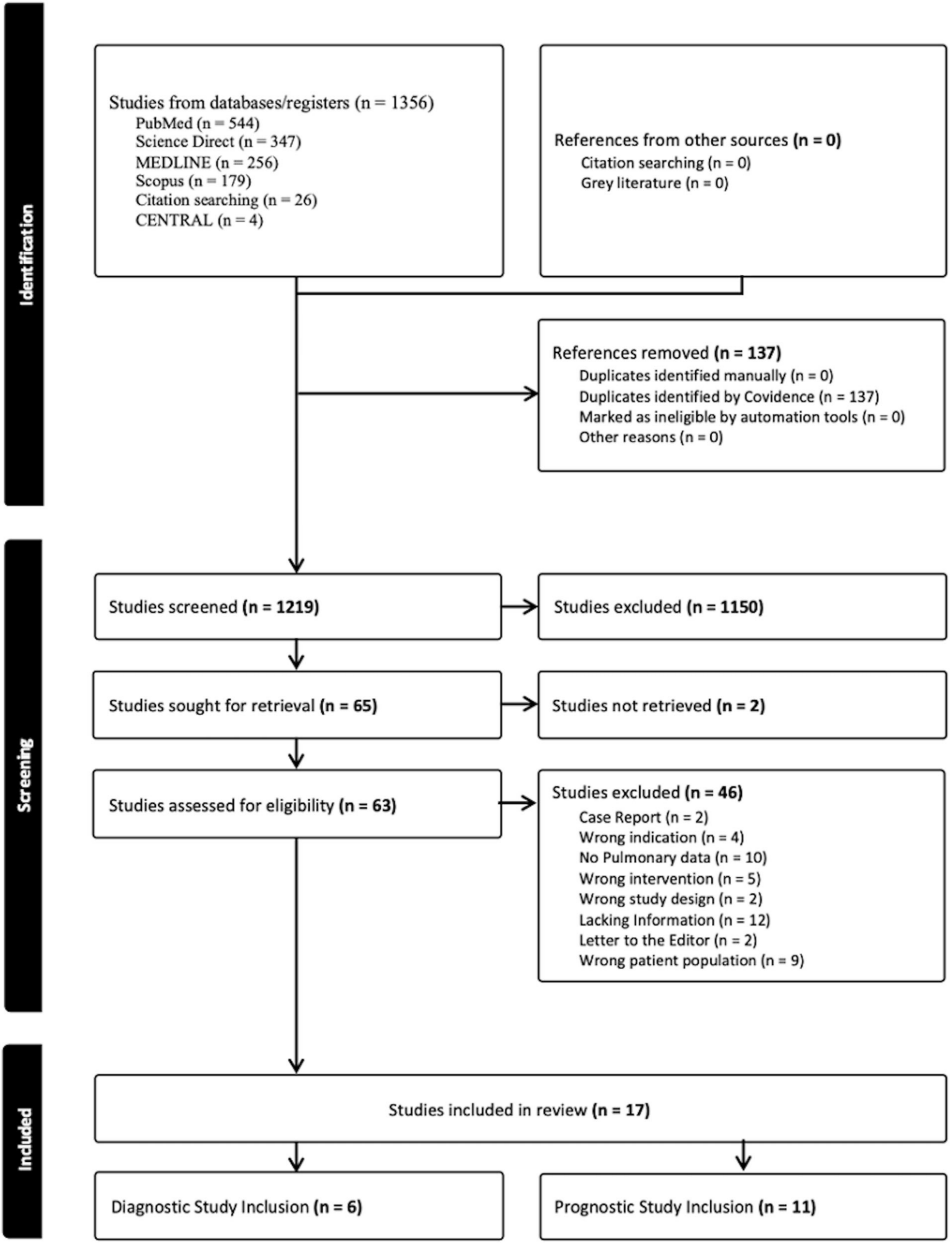
Flow chart of the search for eligible studies on the diagnostic and prognostic performance of [^18^F]FDG-PET/CT or PET/CT for pulmonary sarcoidosis. Publication search concluded with 6 studies included for diagnostic subgroup analysis and 11 studies included for prognostic subgroup analysis

In total, 1355 studies were screened, with 63 deemed eligible for full text review following initial title and abstract screening. In total, 17 studies were deemed appropriate for inclusion. (Fig. 1) Study exclusion following full-text review were due to several reasons including study design, patient cohort and inadequate information relating to pulmonary sarcoidosis. The diagnostic subgroup analysis included 6 studies (Table 1) [11–16]. 11 studies were included in the prognostic section analysis (Table 2) [17–28].

**Table 1:** Summary table of the included studies and participants assessed within the FDG-PET/CT diagnostic subgroup analysis

FDG-PET/CT Diagnostic Subgroup							
Authors/Year	Country	Number	Study	Age (SD)	Diagnosis	Organ Involvement	Methodology
			Design				
Lovinfosse et al [16]	Belgium	169	R	40 (18)	Histological^a^	LP/MH Not Specified	Identification of Sarcoidosis vs HL/DLBCL in tissue proven cases
Braun et al [14]	France	13	R	51	Histological	100% LP	Identification of Biopsy proven sites on PET/CT
Keijsers et al [13]	Netherlands	36	R	39	Histological	94% LP	Identification of Sarcoidosis in histologically proven cases
Keijsers et al [12]	Netherlands	77	R	29 (12.4)	Histological	95% M/H, 71% LP	Identification of Sarcoidosis in histologically proven cases
Maturu et al [15]	India	88	P	43 (11.5)	ATS/ERS/WASOG	99% M/H, 52% LP	Identification of Sarcoidosis in cases of undiagnosed mediastinal lymphadenopathy
Nishiyama et al [11]	Japan	17	R	57 (14.4)	Histological	94% M/H, 22% LP	Comparative 67Ga vs PET/CT sensitivity of biopsy proven sites

American Thoracic Society/European Respiratory Society/World Association for Sarcoidosis and Other Granulomatous Disorders (ATS/ERS/WASOG)

^a^ An undefined majority were diagnosed via histological sampling

Standard Deviation (SD), Retrospective (R), Prospective (P)

Lung Parenchyma (LP), Mediastinal/Hilar (MH)

Each study has been included with their respective organ involvement assessed along with the methodology employed to assess the diagnostic accuracy of FDG-PET/CT

**Table 2:** Summary table of the included studies and participants assessed within the FDG-PET/CT prognostic subgroup analysis

FDG-PET/CT Prognostic Subgroup									
Authors/Year	Country	Number	Study Design	Age (SD)	Diagnosis	SUVmax Site	⍙SUVmax	⍙ FVC	⍙ DLCO
Milman et al [23]	Denmark	10	P	46 (11)	Histological	Lung/Mediastinum	−3.6	1.5	1.8
Vorselaars et al [18]	Netherlands	56	P	48.7 (10.1)	52/56 Histological	Lung Parenchyma	−4.3	6.6	4.1
Saranovic et al [22]	Serbia	30	P	46 (11)	Histological	Lung Parenchyma	−0.87	–	–
Keijsers et al [25]	Netherlands	49	R	43	Histological	Lung Parenchyma	–	12.0	7.0
Chen et al [27]	China	23	R	50 (13)	Histological	Lung/Mediastinum	−8.85	–	–
Maturu et al [24]	India	27	P	47 (10.7)	25/27 Histological	Mediastinum	−6.8	–	–
Umeda et al [28]	Japan	21	P	47.7 (16.5)	Histological	Lung Parenchyma	−5.25	–	–
Schimmelpennink et al [20]	Netherlands	29	R	49.9 (13)	28/29 Histological	Lung Parenchyma	−4.1	8.1	5.1
Schimmelpennink 2019	Netherlands	27	R	48.1 (10)	ATS/ERS/WASOG	Lung Parenchyma	−5.1	4.6	2.4
Keijsers et al [26]	Netherlands	12	P	43.6 (9.3)	Histological	Lung/Mediastinum	−5.4	5.4	3.3
Yakar et al [17]	Turkey	24	P	37.8 (9.3)	Histological	Lung Parenchyma	−6.3	8.6	-0.8

American Thoracic Society/European Respiratory Society/World Association for Sarcoidosis and Other Granulomatous Disorders (ATS/ERS/WASOG)

Standard Deviation (SD), Retrospective (R), Prospective (P)

Each study has been included with their reported change (⍙) in their SUVmax, FVC and DLCO following treatment. The tabulated values represent the change from baseline assessment before and after completion of treatment

### Utility of FDG-PET/CT in diagnosis of sarcoidosis

In the diagnostic subgroup, 6 studies evaluating 400 confirmed sarcoidosis patients were included. There was radiologically proven pulmonary involvement in 392 (95%) of the included patients with 54% males and a mean age of 44.7 years (Table 3). The majority of included studies examined patients with histologically proven de-novo sarcoidosis prior to the commencement of treatment. In the case of Lovinfosse and Nishiyama, an unspecified minority of patients were diagnosed as sarcoidosis based on the ATS/ERS/WASOG criteria [5, 11, 29]. The QUADAS-2 assessment demonstrated some concerns for bias and applicability for the included studies (Supplemental Figs. 1, 2). Bias due to lack of physician blinding to clinical details was deemed to be the greatest area of concern.

**Table 3:** Participant summary characteristic table for both diagnostic and prognostic subgroup analysis

Summary Characteristics Table		
Study	Diagnostic	Prognostic
**Participants**	651 (400 Sarcoid^a^)	308
Age (Mean)	44.7	46
Male	356 (54%)	167 (54%)
Female	295 (46%)	141 (46%)
**Smoking History**	113^b^	122^b^
Current	34	20
Never	54	44
Ex-Smoker	25	58
**Mean Serum ACE**	17.7	53.6
**Sarcoid Involvement**		
Thoracic	392 (95%)	243 (79%)
Lung Parenchyma	62%	60%
Mediastinal/Hilar	92%	86%
Extra Thoracic	152 (38%)	147 (48%)
**Scadding Stage**	147^b^	281^b^
0	7	11
I	51	71
II	72	94
III	6	59
IV	11	46
**Treatment**		
Glucocorticoid Steroids		184
Methotrexate		84
Hydroxychloroquine		15
Leflunomide		1
Azathioprine		13
Infliximab/Biosimilar		112
Adalimumab		10
Indomethacin		8

^a^ 400 Patients were formally diagnosed as Sarcoid from 651 in total

^b^ Representing the number of patients for which this variable was reported

**Fig. 2:**
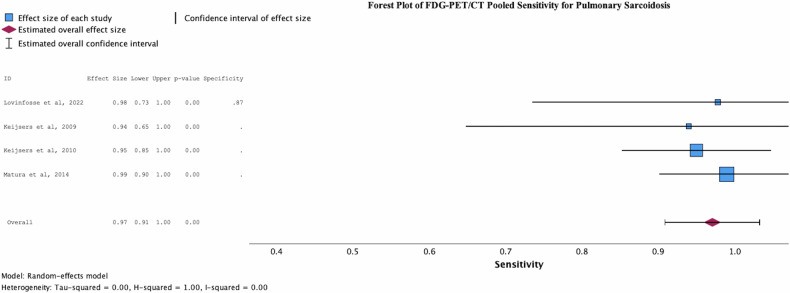
Forest plot demonstrating the observed pooled FDG-PET/CT sensitivity (*n* = 400) in detecting active pulmonary sarcoidosis. The overall effect size generated for the pooled study was 0.971 (Std. Error 0.0316, *p* = < 0.001, 95%CI 0.909–1.000). A heterogeneity score (I^2^) of 0.00 was generated for this plot

The sensitivity of FDG-PET/CT for the diagnosis of pulmonary sarcoidosis was 0.971 (95%CI 0.909–1.000, *p* = < 0.001) (Fig. 2). A single study reported a specificity of FDG-PET/CT in correctly differentiating pulmonary sarcoidosis from lymphoma (*n* = 169) of 0.873 (95%CI 0.845–0.920). The diagnostic odds ratio (DOR) and positive likelihood ratio (LR + ) was 223.57 (95%CI 111.64–447.71) and 7.646 (95%CI 5.70–10.00), respectively. The statistical heterogeneity as, reported by univariate I2 value, was 0.00 for the sensitivity forest plot.

### Utility of FDG-PET/CT in prognostic evaluation of sarcoidosis

A total of 11 studies evaluating 308 patients with confirmed sarcoidosis were included in the prognostic subgroup analysis. Radiologically evidence of pulmonary sarcoidosis was identified in 79% (*n* = 243) of the included population (Tables 2, 3). Of the included patients, 86% were histologically proven, with the remaining being diagnosed via ATS/ERS/WASOG criteria. Patients were distributed across radiographic Scadding Stages, with a total of 11, 71, 94, 59 and 46 classified as Stage 0, I, II, III and IV, respectively. Over 50% of the included patients were treated with glucocorticoid therapy as part of monotherapy, or in combination with other therapies such as methotrexate and hydroxychloroquine. In 36% of cases, patients were treated with infliximab or a biosimilar. Duration of treatment or previous failed treatments were not reported. The QUADAS-2 assessment demonstrated low concern for potential bias and applicability in the 11 included studies (Supplemental Figs. 3, 4).

**Fig. 3:**
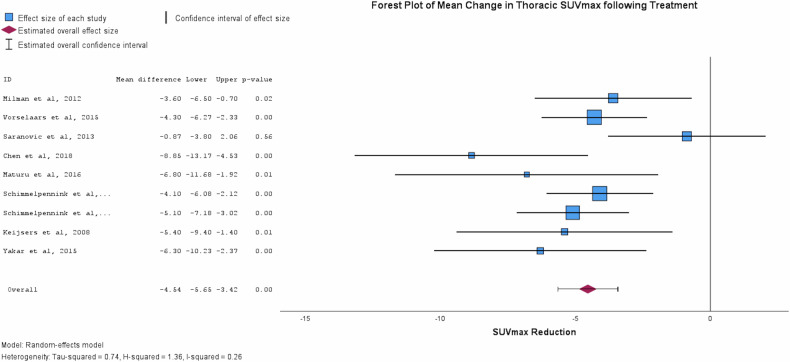
Forest plot demonstrating the observed pooled average ⍙SUVmax (*n* = 259) following completion of treatment from baseline FDG-PET/CT imaging. The pooled observed reduction in SUVmax was 4.54 (95%CI 3.42–5.65). A heterogeneity score (I^2^) of 0.26 was generated for this plot

**Fig. 4:**
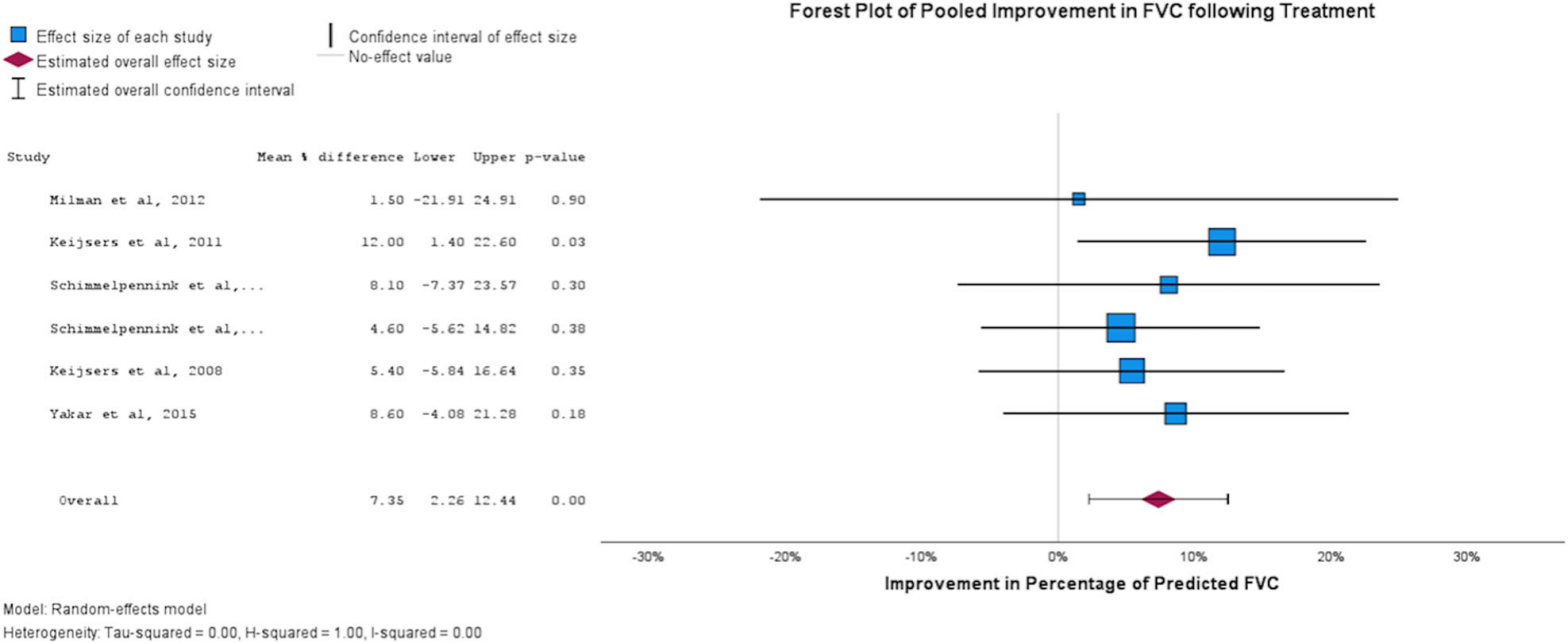
Forest plot demonstrating the observed pooled average ⍙FVC (*n* = 207) following completion of treatment from baseline FDG-PET/CT imaging. ⍙FVC values represent percentage of predicted FVC for each individual. The pooled observed increase in FVC was 7.35% (95%CI 2.26–12.44). A heterogeneity score (I^2^) of 0.00 was generated for this plot

Results of the reported change in SUVmax and pulmonary function are shown in Table 4. A reduction in SUVmax was noted across all the included studies following treatment. Reduction in lung parenchymal and mediastinal station SUVmax as generated by pooled forest and funnel plots were 4.538 (95%CI 5.653–3.453, *p* < 0.001) (Fig. 3, Supplemental Fig. 5) and 3.490 (95% CI 2.446–4.535, *p* < 0.001) (Supplemental Fig. 6), respectively. Pulmonary function improved following treatment; a pooled value for the percentage of predicted FVC and DLCO were 7.346% (95%CI 2.257–12.436, *p* = 0.005) and 3.464% (95%CI 0.205–7.132, *p* = 0.064), respectively (Table 4, Figs. 4/5). Heterogeneity I2 value remained low for all plots, with the highest reported value being 0.26 (Fig. 3). Reduction in lung parenchymal SUVmax strongly correlates with improvement in FVC (r = 0.644, *p* < 0.001) and DLCO (r = 0.582, *p* < 0.001) (Supplemental 7, 9). Scatter plots were generated for eligible studies but failed to identify a strong correlation in the case of FVC (*n* = 46, *r* = 0.005) or the DLCO (*n* = 45, *r* = 0.08) (Supplemental Figs. 8, 10).

**Table 4:** Summary table of mean observed values obtained from forest plot analysis

Forest plot summary table					
				95%CI	
Variable	Effect Size	Std. Error	*p*	Lower	Upper
SUVmax	−4.538	0.5688	< 0.001	−5.653	−3.423
FVC [% Pred]	7.346	2.5967	0.005	2.257	12.436
DLCO [%Pred]	3.464	1.8717	0.064	−0.205	7.132

**Fig. 5:**
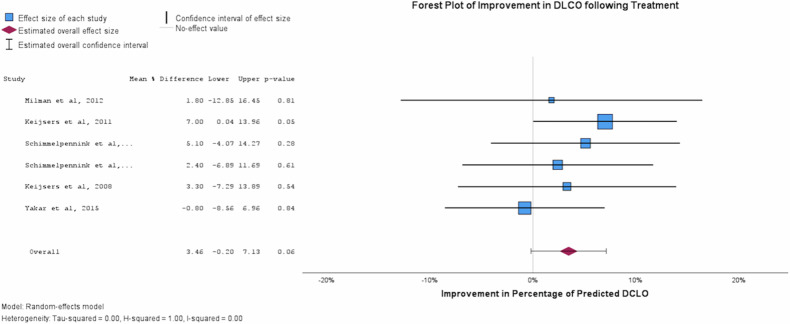
Forest plot demonstrating the observed pooled average ⍙DLCO (*n* = 207) following completion of treatment from baseline FDG-PET/CT imaging. ⍙DLCO values represent the percentage of predicted DLCO for each individual. The pooled observed increase in FVC was 3.46% (95%CI −0.20–7.13). A heterogeneity score (I^2^) of 0.06 was generated for this plot

#### Quality-of-Life Analysis

Of the 17 studies, 4 included a QOL assessment by virtue of a patient-answered questionnaire. The Short Form Health Survey Questionnaire (SF-36) was utilised by 2 studies showing a mean improvement in the respondents subjective scoring (mean 47.15 vs. 37.95, *p* = > 0.05) as per supplemental Table 2. The Sarcoidosis Health Questionnaire (SHQ) was used by 1 study showing no change following treatment. Keijsers reported a binary symptom improvement in 10 of 12 patients following treatment [26]. Overall, QOL indicators as assessed by subjective health scoring systems improved in 75% of the studies assessed (four studies, *n* = 116).

## Discussion

There is little consensus about the role of FDG-PET/CT in pulmonary sarcoidosis [30]. Our aim with this meta-analysis was to provide evidence-based data addressing how functional health outcomes relate to disease activity, as determined by FDG-PET/CT. In addition, we sought to define the diagnostic performance of FDG-PET/CT in cases of suspected pulmonary sarcoidosis [30].

FDG-PET/CT had a sensitivity and specificity of 0.97 and 0.87 in confirming suspected pulmonary sarcoidosis, consistent with those reported in prior literature [14, 30]. There was satisfactory agreement across the included studies with low heterogeneity. Distinguishing malignancy from inflammation on FDG-PET/CT is challenging due to the lack of specificity of FDG as a radiotracer. However, most published literature does not evaluate FDG-PET/CT’s false positive rate, making this difficult to quantify. Lovinfosse assessed the ability of four radiologists to correctly identify pulmonary sarcoidosis on FDG-PET/CT in 420 patients with hypermetabolic thoracic lymph nodes, including sarcoidosis and lymphoma (DLBCL and HL), reporting a specificity of 0.873 [29]. This suggests that FDG-PET/CT may reliably diagnose cases of suspected pulmonary sarcoidosis. There are limitations in the interpretation of a non-dedicated study for confirmation of diagnosis, and selection bias may affect the accuracy of this result. Given that FDG-PET/CT is a relatively source-constrained imaging modality, patients being considered would likely have a higher pre-test probability.

We decided to use SUVmax in assessing PET response to physician-chosen therapy and ultimate functional response. SUVmax is the most commonly used semiquantitative measurement of tracer avidity in a target lesion in clinical PET. It is based on the highest voxel signal intensity within a measured volume and can be affected by various factors including body weight, pharmacodynamics, radiotracer dose, uptake time, scanner type, reconstruction algorithm and acquisition time [31]. Despite its limitations, it remains the most commonly used semiquantitative measurement in clinical PET and has been shown to be a useful disease biomarker in the cardiac sarcoid literature [32]. Our results suggest that SUVmax is an independent variable that changes with treatment. All included studies reported a decrease in SUVmax from baseline, suggesting that it may be a useful imaging biomarker in the assessment of pulmonary sarcoid disease activity following treatment.

When we examined PFT change following treatment, there was an improvement in the percentage of predicted FVC and DLCO by 7.346% and 3.464%, respectively. FVC has long been considered an important functional marker of sarcoidosis disease activity. A decline in FVC, regardless of the objective amount, has been shown to correlate with a relapse or poor response to treatment [33]. However, an ongoing issue with using PFTs to guide treatment is that they do not declare reversible disease and can potentially misclassify efficacious treatments by virtue of their disease phenotype [34]. Moreover, PFTs represent an effort-dependent assessment prone to suboptimal results when patient cooperation and understanding is not achieved. All FDG-PET/CT included studies reported a reduction in SUVmax following treatment. This decrease was associated with an improvement in their functional scores, indicating that FDG-PET/CT observed treatment response may correlate with an improvement in functional health scores. This demonstrates the potential utility of FDG-PET/CT-derived markers of disease severity in cases where uncertainty exists over the reversibility of parenchymal inflammation. In addition, the significant functional improvement in these patients demonstrates the importance of prompt and appropriate treatment to avoid fibrotic transformation in the setting of inflammation [35].

Future studies examining of the role of FDG-PET/CT in sarcoidosis treatment response assessment could be explored using normalised FDG uptake scoring systems (e.g. Deauville in lymphoma and Hopkins’ criteria in lung cancer), allowing for a more reproducible comparison of disease activity [36, 37]. Volumetric PET methods may also provide a more comprehensive lung evaluation. Total lung glycolysis provides a volumetric measurement of regional lung activity [19, 38]. This is yet to be clearly defined and future study is necessary to establish the most accurate PET marker of lung disease progression [19].

In this meta-analysis, we sought to compare PET and functional disease response to patient quality of life scoring systems. In absence of any definitive severity scoring system, patient reported symptom burden remains an important marker of disease remission and relapse. In cases where there was recorded patient response, results were generally positive with 3 of 4 eligible studies demonstrating improvement in patient QOL scores. This may indicate an association between QOL indicators and radiologically observed disease activity which warrants further review.

While the findings of this meta-analysis point to a potential role for FDG-PET/CT in pulmonary sarcoid, it is important to recognise that this is likely beneficial only in selected cases. To our knowledge, no studies have demonstrated a comparative benefit of FDG-PET/CT over the standard CT diagnostic pathway. Indeed, routine CT Thorax with contrast has demonstrated comparable performance to FDG-PET/CT in the cases of stage I/II sarcoidosis—which constitutes the majority in our study cohort. FDG-PET/CT is likely be of most incremental benefit in assessing complex or advanced pulmonary sarcoid (Stage III/IV). However, studies have not yet confirmed its efficacy in this context [39]. Access to PET/CT is another consideration, given the significant differences in availability globally [40]. Future research would be beneficial to define the role of FDG-PET/CT in this particular patient cohort.

### Limitations

Few of the included studies examined QOL indicators in a comprehensive manner allowing for statistical correlation with radiologically assessed disease activity. With a more comprehensive review of symptom burden with standardized questionnaires (e.g. SF-36, SHQ), it may be possible to elucidate more nuanced associations between the location of disease and symptomatology [41].

Patients with established fibrotic versus non-fibrotic sarcoidosis were not clearly defined in this publication. This could have implications on the prognostic improvement that could be expected with immunosuppressive treatment if fibrotic transformation has already taken place. Moreover, the rate of PET/CT defined remission/recurrence following treatment remains poorly understood, with one low-volume study quoting a reduced recurrence in PET/CT responders (14.2% vs 61.5%, *n* = 10) [15]. Further research is needed to examine this relationship.

## Conclusion

FDG-PET/CT has a good diagnostic performance in cases of suspected pulmonary sarcoidosis. FDG-PET/CT variables including relative change in SUVmax appear to correlate with an improvement in pulmonary function. FDG-PET/CT may be a useful adjunct to guide immunosuppression in cases of complex sarcoidosis or where treatment rationalisation is needed.

## Supplementary information

Supplementary Material

## Abbreviations

ATS/ERS/WASOG: American Thoracic Society/European Respiratory Society/World Association for Sarcoidosis and Other Granulomatosis Disorders criteria
DLCO: Diffusion Capacity of the Lungs for Carbon Monoxide
FDG PET/CT: Fluoro-2-deoxyglucose-18 positron emission tomography
FVC: Functional vital capacity
PFT: Pulmonary function test
QOL: Quality-of-Life
SD: Standard Deviation
SUVmax: Standard uptake value maximum
