# Supplementary material for: Meta-analysis of [18F]FDG-PET/CT in pulmonary sarcoidosis
Source: Eur Radiol. 2024 Jul 23;35(4):2222–32. doi: 10.1007/s00330-024-10949-4 (PMC11913913; doi:10.1007/s00330-024-10949-4)
Supplement: Supplementary file 1 — Supplementary Material [file 330_2024_10949_MOESM1_ESM.pdf]

Meta-analysis of 18F-FDG-PET/CT in pulmonary sarcoidosis
ELECTRONIC SUPPLEMENTARY MATERIAL

Reference Standard ATS/ERS/WASOG

The diagnostic accuracy of FDG-PET/CT for thoracic sarcoidosis was compared against the reference standard as defined by ATS/ERS/WASOG[10]. This is non-standardized, but relies on the presence of three major criteria:

1. Presence of non-necrotizing granulomatous inflammation in one or more tissue samples
2. An exclusion of the alternate causes of granulomatous disease
3. Radiological evidence of disease

The reference standard was kept purposely broad in this case, given the relative paucity of information that is currently available in the subject area. A preference was given to studies which had a histological proven diagnosis.

Supplemental Diagnostic Subgroup Risk of Bias Assessment:

One key area of potential bias in this subgroup was deemed be related to patient selection and the lack of physician blinding. In the case of patient selection, methods of patient recruitment were often not clearly outlined. Few authors outlined a consecutive or random sampling of patients and were unclear on exclusion criteria. Concerns in the domain of the index test arose in 3 of the included studies which did not blind the reporting physicians to the clinical data or previous imaging. This would have obvious implications on the comparison of imaging modalities for diagnostic purposes. Indeed, a risk of bias were also identified in flow and timing given that studies did not identify the time from diagnosis to the index test.

Supplemental Prognostic Subgroup Risk of Bias Assessment:

All studies demonstrated a low risk of bias in the domains of patient selection. A key area of potential bias was identified to be the index test domain. None of the included studies clearly identified whether interpreters of the index test had been blinded to the reference standard or clinical details of the patient. Lack of blinding to these details could have led to a reporting bias and affected the overall impression of disease activity.

Supplemental Figure 1:
Diagnostic subgroup (n = 6) risk of bias traffic light assessment using the QUADAS-2 tool.

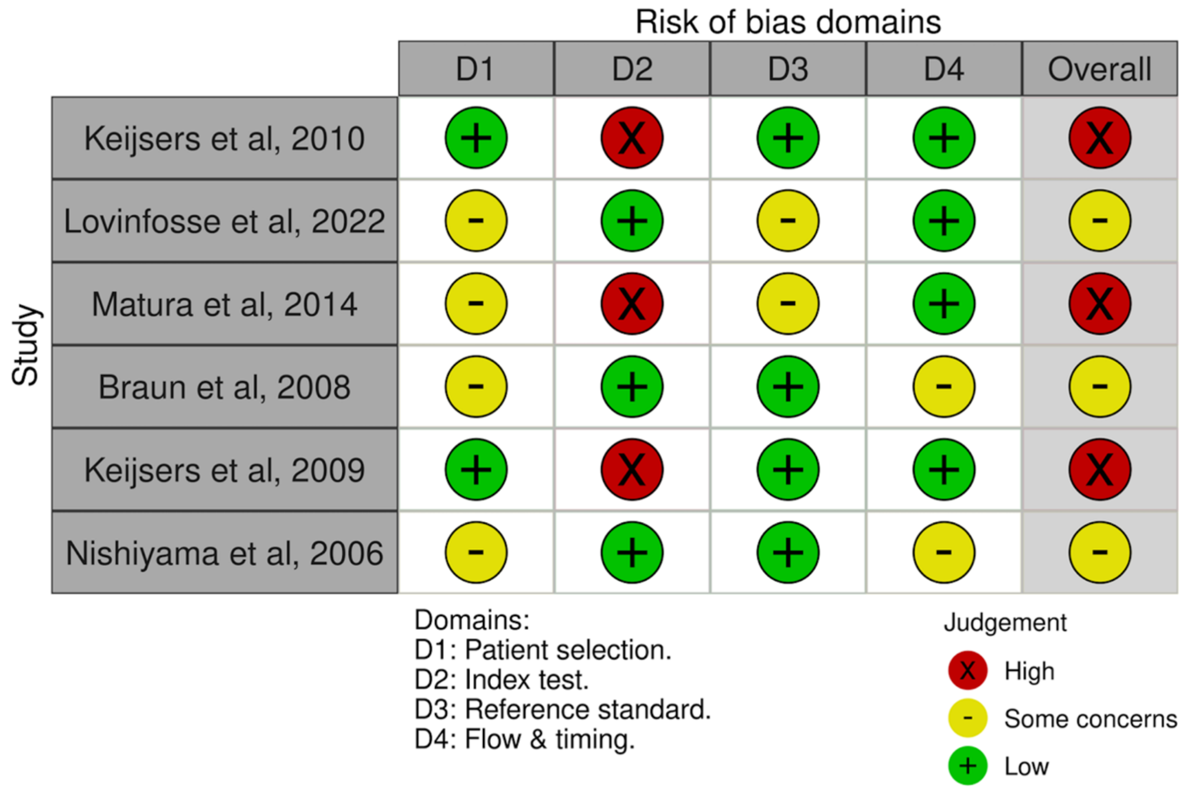

Supplemental Figure 2:
Diagnostic subgroup population-weighted risk of bias assessment using the QUADAS-2 tool.

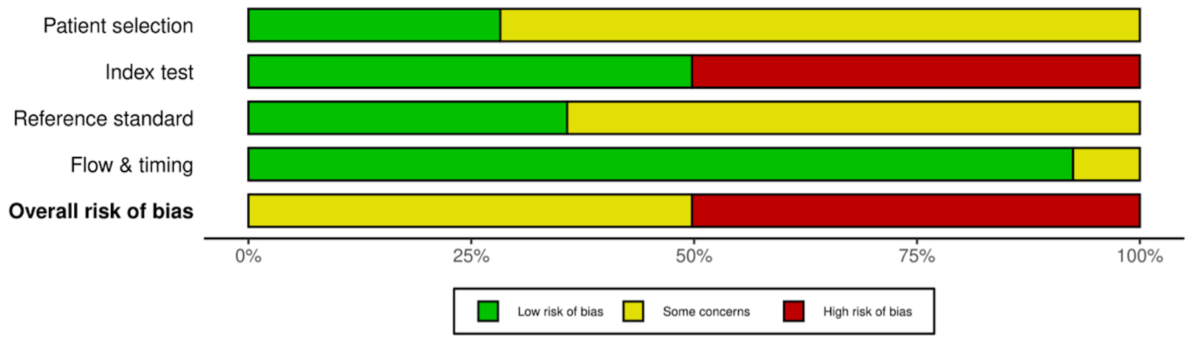

Supplemental Figure 3:
Prognostic subgroup (n = 11) risk of bias traffic light assessment using the QUADAS-2 tool.

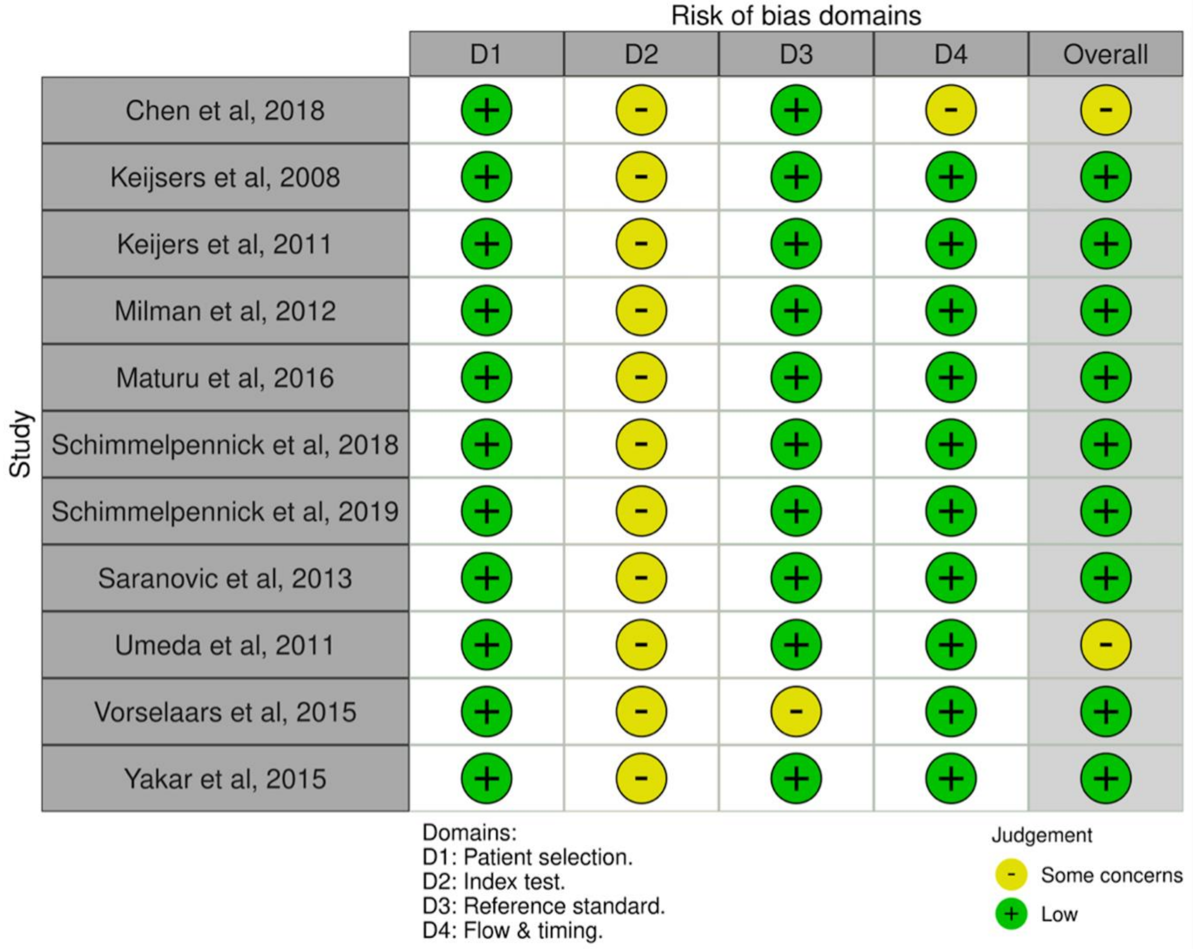

Supplemental Figure 4:
Prognostic subgroup population-weighted risk of bias assessment using the QUADAS-2 tool.

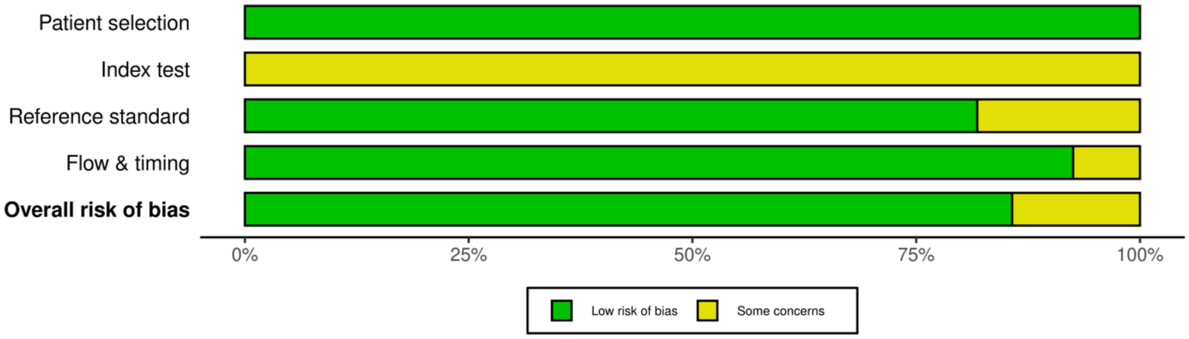

Supplemental Figure 5
Funnel plot assessment of the discrete lung parenchymal ∆SUVmax values from each study as compared to the observed pooled average (as generated by forest plot) and 95% confidence intervals.

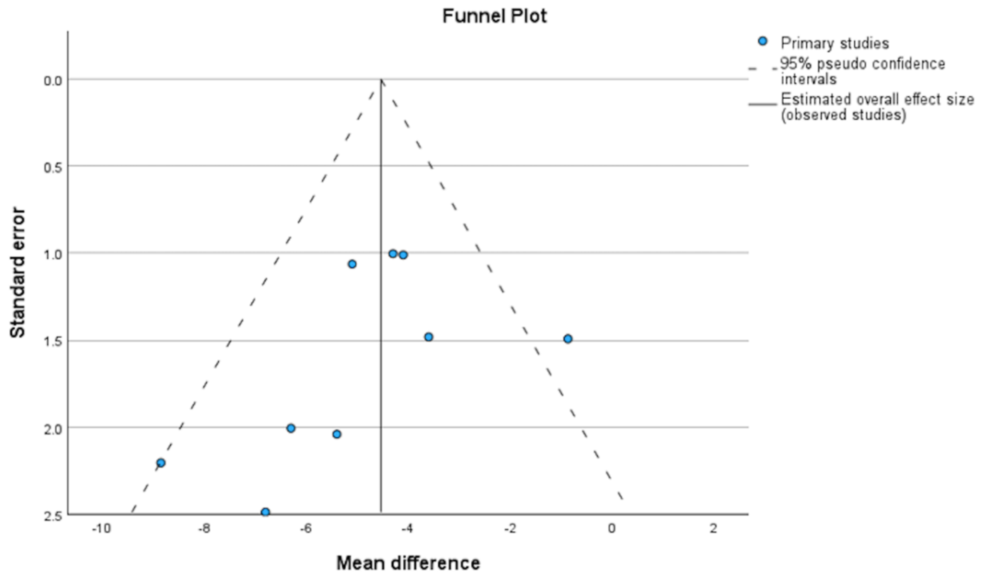

Supplemental Figure 6
Forest Plot of pooled ∆SUVmax for mediastinal stations following treatment.

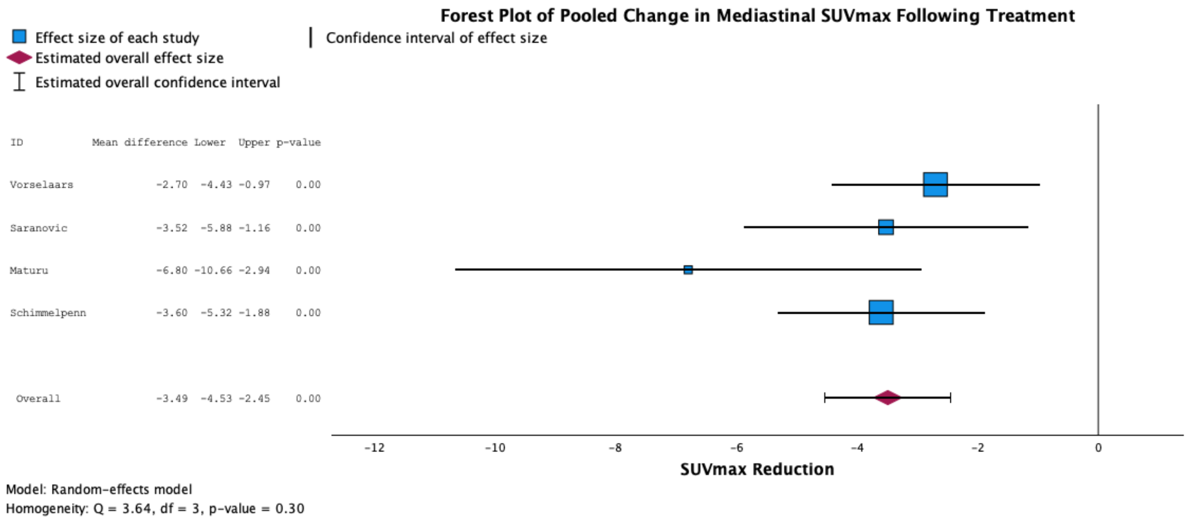

Supplemental Figure 7:
Funnel plot assessment of the discrete ∆FVC values from each study as compared to the observed pooled average (as generated by forest plot) and 95% confidence intervals.

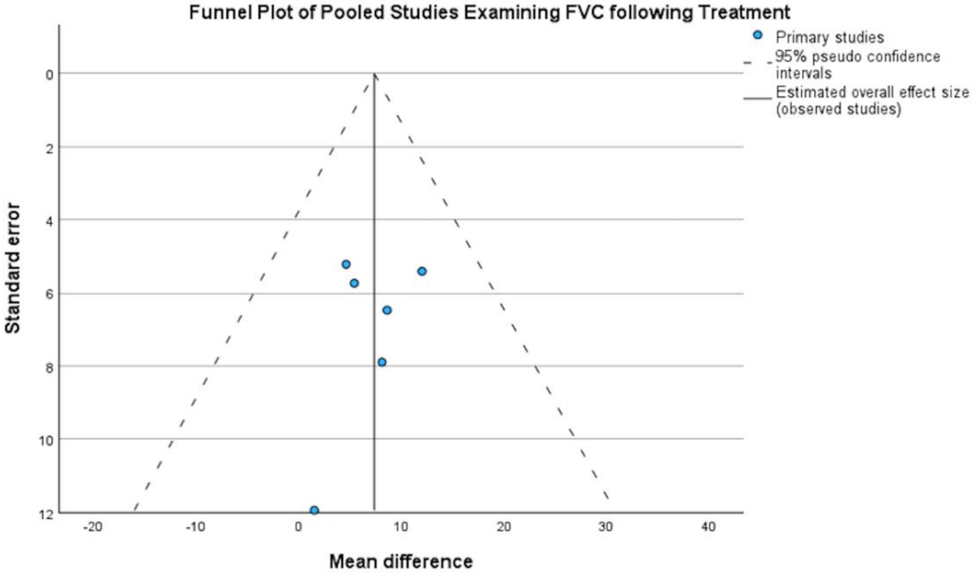

Supplemental Figure 8:
Graphical pairwise correlation of discrete ∆FVC values and ∆SUVmax values following treatment (n = 46, R^2 = 5.849^{-7}). ∆FVC values represent percentage of predicted for each individual.

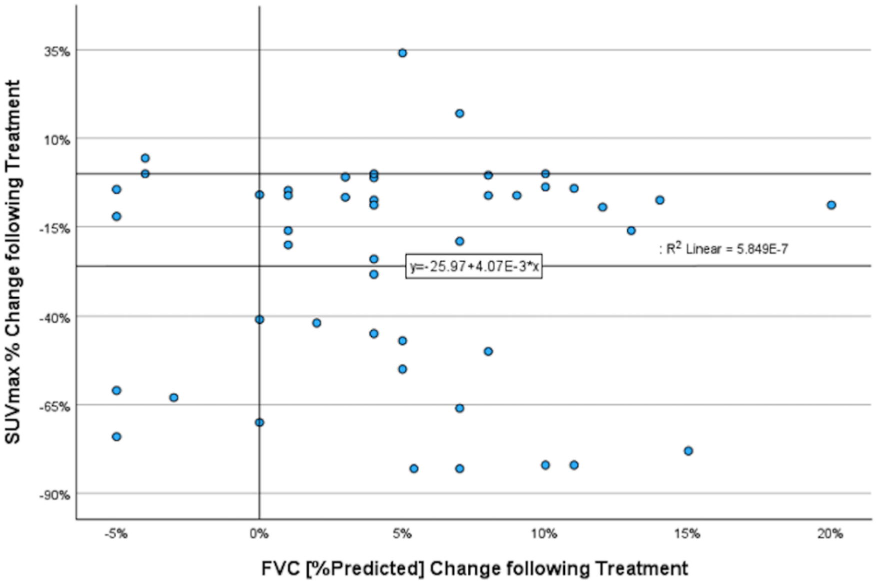

Supplemental Figure 9:
Funnel plot assessment of the discrete ∆DLCO values from each study as compared to the observed pooled average (as generated by forest plot) and 95% confidence intervals.

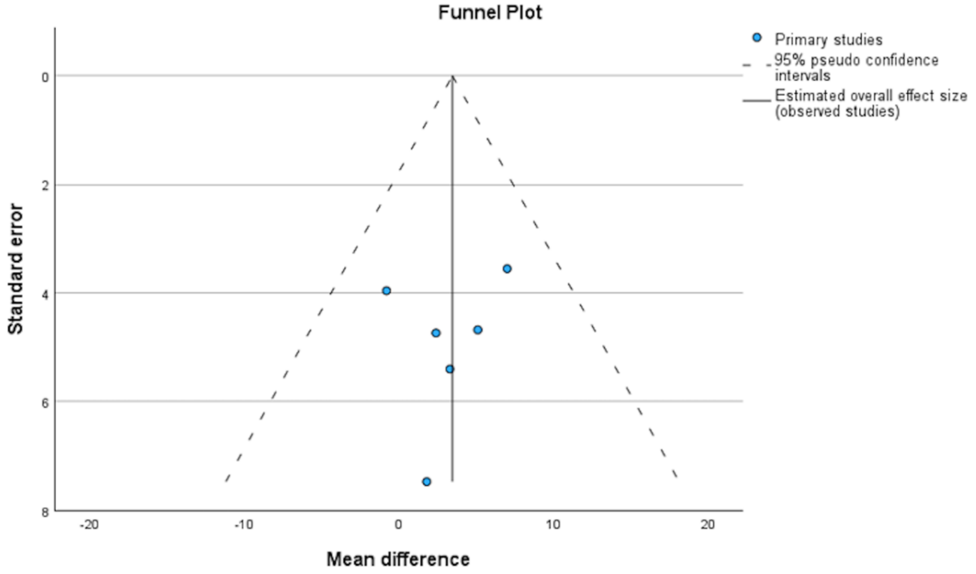

Supplemental Figure 10:
Graphical pairwise correlation of discrete ∆DLCO values and ∆SUVmax values following treatment (n = 45, R^2 = 0.080). ∆DLCO values represent percentage of predicted for each individual.

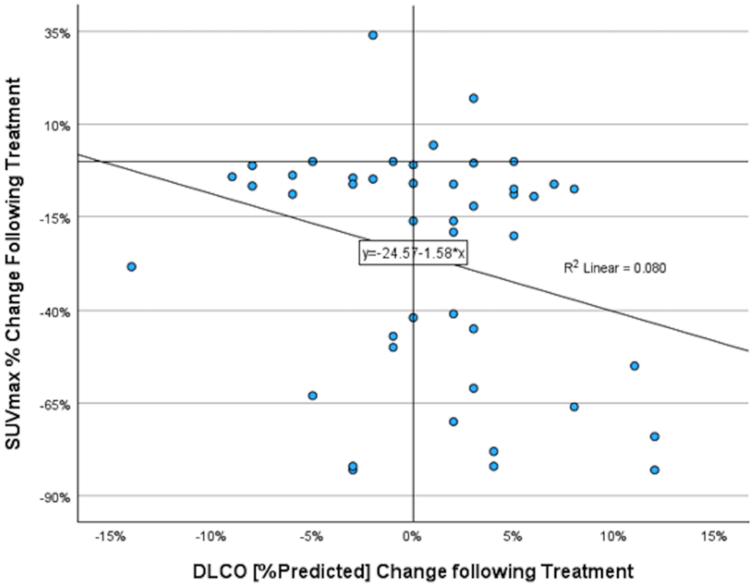

Supplemental Table 1:
Pairwise correlation analysis of ∆pulmonary function scores and ∆SUVmax using Pearson Correlation Coefficient (r).

Variable 1	Variable 2	Pearson Correlation (r)
∆SUVmax (Lung)	∆ FVC	0.644 (p < 0.000)
∆SUVmax (Lung)	∆ DLCO	0.582 (p < 0.000)

Supplemental Table 2:
Quality-of-Life Scoring Assessment Summary.

Quality-of-Life Summary Table

Study	Scoring System	Participants	Post-Treatment Score Mean (SD)	Pre-Treatment Score Mean (SD)
Vorselaars 2015	Health SF-36	48	48.8	40.6
Maturu 2016	SHQ	27	5.1 (0.6)	5.1 (0.6)
Schimmelpennink 2018	Health SF-36	29	45.5 (28.1)	35.3 (21.6)
Keijsers 2008	Symptom Burden*	12	10 Improved	All Symptomatic
